# Genome Sequencing of Ancient Plant Remains: Findings, Uses and Potential Applications for the Study and Improvement of Modern Crops

**DOI:** 10.3389/fpls.2018.00441

**Published:** 2018-04-17

**Authors:** Antimo Di Donato, Edgardo Filippone, Maria R. Ercolano, Luigi Frusciante

**Affiliations:** Department of Agricultural Sciences, University of Naples Federico II, Portici, Italy

**Keywords:** ancient DNA, next-generation sequencing, crop breeding, genomics, domestication

## Abstract

The advent of new sequencing technologies is revolutionizing the studies of ancient DNA (aDNA). In the last 30 years, DNA extracted from the ancient remains of several plant species has been explored in small-scale studies, contributing to understand the adaptation, and migration patterns of important crops. More recently, NGS technologies applied on aDNA have opened up new avenues of research, allowing investigation of the domestication process on the whole-genome scale. Genomic approaches based on genome-wide and targeted sequencing have been shown to provide important information on crop evolution and on the history of agriculture. Huge amounts of next-generation sequencing (NGS) data offer various solutions to overcome problems related to the origin of the material, such as degradation, fragmentation of polynucleotides, and external contamination. Recent advances made in several crop domestication studies have boosted interest in this research area. Remains of any nature are potential candidates for aDNA recovery and almost all the analyses that can be made on fresh DNA can also be performed on aDNA. The analysis performed on aDNA can shed light on many phylogenetic questions concerning evolution, domestication, and improvement of plant species. It is a powerful instrument to reconstruct patterns of crop adaptation and migration. Information gathered can also be used in many fields of modern agriculture such as classical breeding, genome editing, pest management, and product promotion. Whilst unlocking the hidden genome of ancient crops offers great potential, the onus is now on the research community to use such information to gain new insight into agriculture.

## Introduction

Over time, important plant families such as the Poaceae, Solanaceae, Fabaceae, and Cucurbitaceae have been domesticated for human needs. Agriculture has had a dramatic impact on human migration and settlements, providing access in most cases to a reliable food supply. Those who through biogeographical good fortune first acquired domesticates gained enormous advantages over other peoples and were able to expand their sphere of influence rapidly (Vinet and Zhedanov, 2010).

Current knowledge of plant domestication is largely derived from morphological analysis of archeological and herbarium remains and/or population genetic analysis of present-day samples. Tracing the domestication history of a species can provide insights into the selection of important traits, facilitating both the use of genetic resources and the management of germplasm repositories (Blanca et al., 2015). The domestication process has led to favorable phenotypic changes in traits such as fruit, seeds or tubers in the genetic makeup of ancestral wild species. For instance, enlarged fruit size was selected during domestication whilst other traits were eliminated. However, recovering wild ancestor alleles can still improve the productivity of many crops (Soyk et al., 2017). Genetic studies of ancient plants allow us to reconstruct the pattern of gene distribution in an area as well as the gene introgression process in modern crops. Indeed, species continually incorporate varying degrees of population admixture, reassembling themselves.

Small-scale aDNA studies can help to reveal patterns of crop adaptation and migration. However, they do not permit investigation of the impact of such events on whole crop genomes. For this reason, whole-genome scale studies on ancient genomes have been conducted in recent years, paving the way for many future studies in this fascinating field of research.

## Looking for ancient plant DNA

In the last 30 years, DNA has been extracted from several ancient biological remains and substrates most frequently studied in palaeogenetic research. Since the first successful attempts to extract ancient DNA from horses in the 1980s (Higuchi et al., 1984), plant aDNA has been obtained from different types of biological material and/or artifacts (Table 1).

**Table 1:** A collection of aDNA studies on crop remains sorted by tissue type.

**Species^a^**	**Common name^b^**	**Tissue^c^**	**Deposit or material^d^**	**Age^e^**	**Kind of study^f^**	**References^g^**	**aDNA extraction method^h^**
*Triticum* sp.	Wheat	Charred seed	Archaeobotanical remains	8400 BC−700 AD	nuDNA amplification and sequencing	Bilgic et al., 2016	Bilgic et al., 2016
*Lagenaria siceraria*	Bottle gourd	Fruit	Archaeobotanical remains	10000 BP	Genotype assignment through molecular markers	Erickson et al., 2005	Goloubinoff et al., 1993
*Cucurbita* sp.	Squash	Fruit and peduncle	Archaeobotanical remains	10000–0 BP	ptDNA region amplification and sequencing	Kistler et al., 2015	Kistler, 2012
*Prunus* sp.	Plum	Fruit stone	Archaeobotanical remains	2000 BP	ptDNA region amplification and sequencing	Pollmann et al., 2005	Höss and Pääbo, 1993; Pollmann et al., 2005
*Olea* sp.	Olive	Fruit stone	Archaeobotanical remains	5500–4500 BP	rDNA region amplification and sequencing	Elbaum et al., 2006	DNeasy Plant Mini kit (Qiagen, Valencia, CA, USA; Schlumbaum et al., 1998)
*Citrullus* sp.	Watermelon	Leaf	Herbarium specimens	177 BP	ptDNA, nuDNA region amplification, and sequencing	Chomicki and Renner, 2015	Plant DNA extraction kit (NucleoSpin; Macherey–Nagel, Duren, Germany)
*Arabidopsis thaliana*	Thale cress	Leaf	Herbarium specimens	87–0 BP	Genome sequencing	Exposito-Alonso et al., 2016	Yoshida et al., 2013
*Hesperelaea palmeri*	–	Leaf	Herbarium specimens	75 BP	ptDNA, rDNA region amplification, and sequencing	Zedane et al., 2016	DNeasy Plant Mini kit (Qiagen, Valencia, CA, USA)
*Pinus sylvestris, Picea abies*	Scots pine, Norway spruce	Pollen	Lake sediments	100–10000 BP	ptDNA region amplification and sequencing	Parducci et al., 2005	DNeasy Plant Mini kit (Qiagen, Valencia, CA, USA)
Many plant genera	Many genera	Pollen	Animal rumen contents	10500 BP	ptDNA region amplification and sequencing	Van Geel et al., 2014	Van Geel et al., 2014
Many genera and species, especially *Ipomoea* sp.	Sweet potato	Seed and piece of leaf	Lake sediments	5000–0 BP	ptDNA region amplification and sequencing	Bremond et al., 2017	Bremond et al., 2017
*Chenopod* sp.	Chenopod	Seeds	Archaeobotanical remains	4000 BP	ptDNA region amplification and sequencing	Kistler and Shapiro, 2011	DNeasy Plant Mini kit (Qiagen, Valencia, CA, USA)
*Panicum* sp.	Panic grass	Seeds	Archaeobotanical remains	7900–7400 BP	ptDNA region amplification and sequencing	Fornaciari et al., 2018	Kistler and Shapiro, 2011
*Gossypium* sp.	Cotton	Seeds	Archaeobotanical remains	3850–750 BP	Genome sequencing	Palmer et al., 2012	Palmer et al., 2012
*Vitis* sp.	Grape vine	Seeds	Archaeobotanical remains	4000 BP	Targeted sequencing of ptDNA and nuDNA	Wales et al., 2016	Manen et al., 2005; Wales et al., 2014
*Hordeum* sp.	Barley	Seeds and spikelet	Archaeobotanical remains	6200–5800 BP	Exome sequencing	Mascher et al., 2016	Kistler, 2012
*Zea mays*	Maize	Spikelet	Archaeobotanical remains	5310 BP	Genome and targeted sequencing	Ramos-Madrigal et al., 2016	Ramos-Madrigal et al., 2016
*Olea europea, Origanum vulgare* and other genera	Olive, oregano and others	Unknown	Ancient pottery	4350 BP	ptDNA region amplification	Hansson and Foley, 2008	Hansson and Foley, 2008
Many taxa	–	Unknown	Ancient herbivore middens	30490–710 BP	ptDNA region amplification and sequencing	Murray et al., 2012	Haile, 2012
Many plant families	Many plant families	Unknown	Cave sediments	400000–50 BP	ptDNA region amplification and sequencing	Willerslev, 2003	Willerslev, 2003
Many plant families	Many plant families	Unknown	Palaeofaeces	2000 BP	ptDNA region amplification and sequencing	Poinar et al., 2001	Poinar, 1998
Many plant genera	Many genera	Unknown	Human gut contents	5000 BP	ptDNA region amplification and sequencing	Rollo et al., 2002	Rollo et al., 2002
*Abies* sp. *Pinus* sp. *Fagus* sp. *Quercus* sp.	–	Wood	Archaeobotanical remains	11500–300 BP	ptDNA region amplification and sequencing	Liepelt et al., 2006	Plant DNA Mini Kit (Qiagen, Germany)

a *Species, genera, or general taxa assigned to samples analyzed in the work*.

b *Common name of crops most related to the analyzed sample*.

c *Tissue used for aDNA extraction*.

d *Material or deposit*.

e *Age of sample reported in the work in year Before Present (BP) or in Gregorian date format*.

f *Information regarding the kind of genetic study conducted*.

g *Reference regarding the work*.

h *Reference or indication regarding the method of DNA extraction used in the work*.

Seeds are among the most highly prized sources of aDNA, especially when charred, desiccated, frozen, or deposited in anoxic conditions (Green and Speller, 2017). Seeds of wheat (Bilgic et al., 2016), barley (Mascher et al., 2016), cotton (Palmer et al., 2012), grapevines (Wales et al., 2016) and other crops have been found to contain DNA that can shed light on the origin, evolution and domestication of age-old crops. In addition to seeds, the DNA of ancient spikelets and combs (Mascher et al., 2016; Ramos-Madrigal et al., 2016) has also been analyzed. Successful aDNA extraction was even obtained from fruit, especially from lignified material such as fruit stones, rind, and peduncles (Pollmann et al., 2005; Elbaum et al., 2006; Kistler et al., 2015). The ancient wood structure of plant remains, such as residues present on building components and on utensils, residues left during plowing, harvesting, transformation, storage, and transport of crops, was also used for genetic analysis (Liepelt et al., 2006). aDNA fragments inside 2,400-year-old Classical Greek amphoras were amplified although in the starting material there was no trace of plant residues under naked-eye examination (Hansson and Foley, 2008). Another important source of aDNA consists in lake and cave sediments, where several kinds of ancient plant remains can be found. The geological context of lakes provides a robust archive for the retrieval of ancient plant DNA through time and reflects the effect of all environments worldwide (Willerslev, 2003; Bremond et al., 2017; Parducci et al., 2017). Plant residues can also be found in ancient animal and human remains such as palaeofaeces, hair, dental calculus, and gastrointestinal contents (Poinar et al., 2001; Rawlence et al., 2014; Van Geel et al., 2014; Weyrich et al., 2015).

Recently, herbarium archives have demonstrated their long-term genetic potential through successful recovery of aDNA from historic plant collections (Chomicki and Renner, 2015; Exposito-Alonso et al., 2016; Zedane et al., 2016), probably constituting the best conserved and most abundant resources in the modern era (Bakker, 2017; Green and Speller, 2017).

## The process of aDNA extraction and authentication

Studies conducted on ancient plant DNA use different extraction techniques (Table 1), standard procedures being modified according to the starting material in question. Commercially available DNA extraction kits, with key modifications, have proved to be very efficient in recovering ancient plant DNA (Parducci et al., 2005; Elbaum et al., 2006; Liepelt et al., 2006; Kistler and Shapiro, 2011; Chomicki and Renner, 2015; Zedane et al., 2016). Protocols based on cetyltrimethylammonium bromide (CTAB) were adapted for more difficult samples (Pollmann et al., 2005; Bilgic et al., 2016; Fornaciari et al., 2018). Silica-based extraction methods also proved successful in many cases (Rollo et al., 2002; Palmer et al., 2012; Van Geel et al., 2014). Identifying the most efficient DNA extraction method is crucial since DNA yield and quality can vary considerably depending on the substrates and the preservation conditions. All ancient tissues or substrates contain a small amount of endogenous DNA, and the quality of the DNA is very poor due to the large number of post-mortem mutations occurring (Carpenter et al., 2013). Moreover, present-day human and bacterial contaminations are inevitably introduced during excavation, preservation and laboratory work (Gansauge and Meyer, 2014). The use of non-efficient extraction methods could increase the likelihood of recovering very limited, degraded and/or contaminated DNA (Threadgold and Brown, 2003). A well-calibrated combination of DNA extraction and purification steps is necessary to prevent further degradation of the already damaged and fragile ancient nucleic acid. Suitable methodologies should maximize the recovery of good quality aDNA from ancient plant specimens and minimize co-extraction of other DNA as well as substances that inhibit PCR. Non-destructive and non-invasive sampling methods have been developed and implemented in order to maintain the integrity of archaeobotanical samples and store sufficient material for further analysis (Green and Speller, 2017). Precise cataloging and characterization of archaeobotanical remains can lead to improvements in genotype and phenotype authentication of ancient organisms. A wide range of analytical approaches can be used to both complement and validate ancient genetic information, including microscopy, lipid analysis, proteomics, metabolomics, radiocarbon dating, collagen peptide mass fingerprinting, and bioinformatics (Green and Speller, 2017). In particular, bioinformatic approaches and molecular methodologies may improve the process of obtaining information from minute samples.

## From molecular markers to sequencing technologies

In recent years, the methodologies used in aDNA investigation have changed enormously, providing an even better understanding of the genetic diversity of crop species over time and space. The development of polymerase chain reaction (PCR) and of PCR-derived molecular markers in the 1980s proved to be crucial for early aDNA analysis. Most aDNA phylogenetically informative studies concern the DNA amplification of specific organelles such as the plastids. Ribosomal DNA (rDNA) genes are also of interest for aDNA research (Elbaum et al., 2006; Zedane et al., 2016), whereas plant mitochondrial (mtDNA) studies are rarer in plant aDNA research. Organelle nucleotide regions are conserved among plant organisms, greatly simplifying the design of primers, amplification of target sequence and the Sanger sequencing of small fragments (Schlumbaum et al., 2008). Moreover, aDNA, which by its very nature is extremely degraded, often damaged, and typically short and fragmented, is better preserved in organelle genomes where it exists in multiple copies per cell. Over the years researchers have developed advanced molecular technologies for investigating ancient nuclear DNA (nuDNA) since it carries several important loci. Genetic studies on archaeobotanical remains have been conducted using nuclear sequences or markers based on important genes related to agronomic traits (Blatter et al., 2002; Freitas et al., 2003; Jaenicke-Despreés, 2003). NuDNA is also more susceptible to degradation, and some polynucleotides are more damaged than others (Weiß et al., 2016). For instance, substitutions resulting from deamination cytosine residues are vastly over-represented in aDNA sequences. Miscoding of C to T and G to A accounts for the majority of errors (Gansauge and Meyer, 2014).

The development of massive parallel DNA sequencing, also coupled with enriched capture-based methods, has improved many critical issues of aDNA research (Green and Speller, 2017). The generation of gigabases of data through next-generation sequencing (NGS) technologies has overcome many of the limits of the previous methodologies, allowing huge genomic regions or whole genomes to be covered. The number of reads that can be processed in aDNA analyses is constantly increasing thanks to new NGS technologies that can achieve 1.8 billion reads in one run (Yin et al., 2017). NGS produces large numbers of short sequencing reads, which is particularly useful for aDNA analysis for its fragmentation and degradation (Gutaker and Burbano, 2017).

New bioinformatics tools, protocols and studies have been released to improve efficiency in analysing genomic aDNA data (Binladen et al., 2006; Kistler et al., 2017). The sequencing errors can be resolved, for example, by trimming some bases from the 5′-end of reads, filtering contamination-derived reads, and reducing the number of mismatched bases for mapping reads (Schubert et al., 2012).

However, the use of true single molecule and nanopore sequencing methods on ancient polynucleotides is currently under discussion (Hofreiter et al., 2015). Indeed, the fragmented structure of damaged aDNA molecules could make the use of PacBio and Oxford Nanopore very difficult because these technologies produce long reads and currently suffer from high error rates (Laver et al., 2015; Rhoads and Au, 2015).

The “impossible genome” (Der Sarkissian et al., 2015) of ancient crops or species related with modern crops is now accessible, enabling the study of complex agronomic traits. Ancient whole-genome sequencing with modern NGS technologies were successfully conducted in recent years on major crops, namely cotton and maize (Palmer et al., 2012; Ramos-Madrigal et al., 2016), and other important plant species (Exposito-Alonso et al., 2016). Not all samples can be analyzed using whole shotgun sequencing since assembling complete plant genomes is a major challenge even for modern samples due to their large, highly repetitive and heterozygous genomes and varying ploidy levels (Der Sarkissian et al., 2015).

Target hybridization enrichment technology provides an approach to enrich a DNA pool for large genomic regions, such as genes, exomes, organelle genomes, and even whole genomes. This technique is useful to capture target DNA of interest and discriminate exogenous polynucleotides (Di Donato et al., 2017). aDNA of maize and of barley exomes has been captured and sequenced (Mascher et al., 2016; Ramos-Madrigal et al., 2016), paving the way for other targeted sequencing on ancient crop remains.

## Analysis of aDNA genomic data

Sequences and other information from aDNA can be used in different ways depending on the research aims. Almost all of the analyses that can be performed on fresh DNA are also possible on aDNA (Supplementary Figure 1). DNA barcoding is useful to identify species, genera or families, using diagnostic variation in a suitable DNA region (Sonstebo et al., 2010). Recent NGS advances have boosted research interest in this methodology, especially for its metagenomic application on lake sediments and other complex materials (Murray et al., 2012; Leonardi et al., 2016; Parducci et al., 2017).

The availability of DNA from ancient plants allows phylogenetic analysis between ancient and modern samples to be inferred. In recent years “omics” approaches have produced an enormous amount of data on hundreds of plant species, especially crops, making phylogenetic analysis on aDNA increasingly effective. Indeed, land plant genetic distance and evolution studies and Angiosperm Phylogeny Group classification (APG) have been improved thanks to several plant phylogenetic studies (Chase et al., 2016). Within such approaches, aDNA can solve many phylogenetic questions concerning the evolution, domestication and improvement of plant species. Phylogenetic studies based on genetic markers have already successfully highlighted the genetic correlation between ancient and modern samples (Kistler and Shapiro, 2011). However, such studies are not exhaustive because they only analyse a small part of plant genomes. Hence, the latest challenge for aDNA studies is phylogenomic analysis. Indeed, specific bioinformatic suites have been developed to reconstruct ancient genomes (Orlando et al., 2015).

Thanks to NGS technologies and the development of new statistical approaches for detecting and quantifying admixture from genomic data, previously unknown hybridization events between living organisms have been revealed (Schaefer et al., 2016). Historically aDNA studies were used to identify relationships between species or populations and to discriminate genotypes in widely distributed populations of maize (Ramos-Madrigal et al., 2016) and barley (Mascher et al., 2016). with the aid of aDNA admixture-based approaches.

## The application of aDNA genome sequencing for modern crop improvement and promotion

The information obtained from aDNA studies can be applied in modern agriculture and various fields of research. Knowledge of mechanisms and rates of evolution of land plants can be directly achieved through experiments with both modern and ancient samples (Gutaker and Burbano, 2017).

Ancient genomics can provide insights into plant-pathogen interactions, revealing details about the coevolution of crops and pathogens, with implications for modern crop breeding and management. For example, DNA analysis of historical herbarium specimens showed that the strain of *Phytophthora infestans* involved in the nineteenth century Irish potato famine differs from all examined modern strains (Yoshida et al., 2013). A study of ancient genomes revealed a gene flow between cultivated and sympatric wild populations of barley crops over 6,000 years ago, supported by phylogeographic data (Mascher et al., 2016). Palaeo-ecological reconstructions over thousands of years can be conducted from aDNA extracted from lake and cave sediments. The sediment material created and stratified year after year illustrates the history of species in a given area, evidencing patterns of trade and migration, ecosystem and agroecosystem changes. For instance, through meta-barcoding studies on lake sediments it was possible to trace the introduction and history of agriculture in Benin, detecting when the sweet potato (*Ipomoea* sp.) was introduced into the region (Bremond et al., 2017).

**Figure 1:**
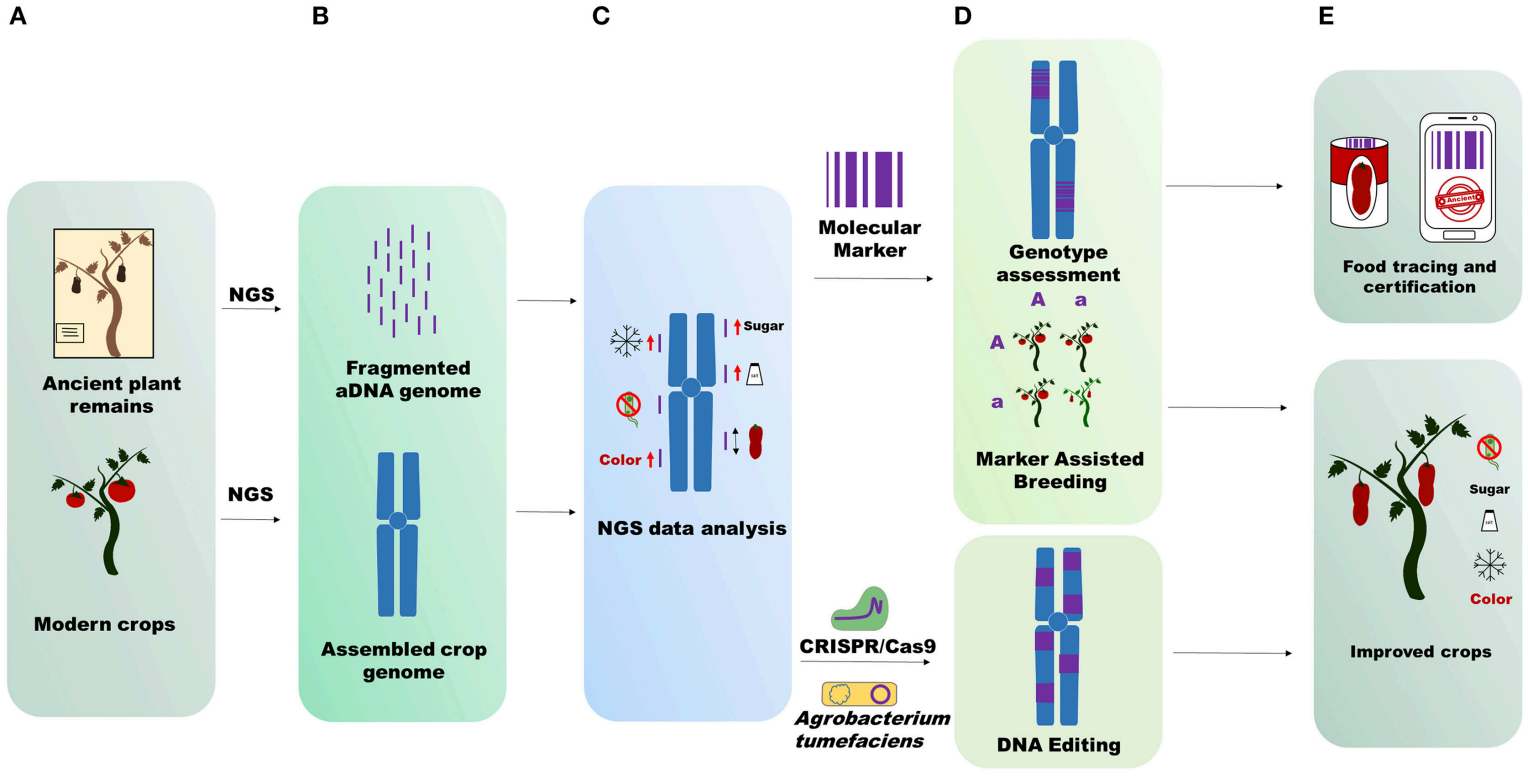
Applications of ancient genome sequencing. **(A)** Starting material for NGS sequencing. **(B)** Upper part, aDNA short fragmented sequences difficult to assemble; bottom part, modern crop genomes assembled in pseudomolecules (chromosomes). **(C)** NGS data analysis. aDNA mapping on the reference crop genome identifies structural variants that influence some importance agricultural traits. Icons represent fruit sweetness, flavor, long fruit, color, resistance to abiotic, and biotic stress. **(D)** Techniques unlocked through aDNA genome sequencing. Molecular marker design on ancient sequences for genotype assessment or for crop breeding; Identification of new targets for genetic transformation by *Agrobacterium tumefaciens* or genome editing by CRISP/Cas9. **(E)** aDNA genome sequencing data output utilization. Analyses conducted on aDNA genomes are useful for food tracing and certification (molecular marker) and for improvement of modern crops (DNA editing and Marker Assisted Breeding).

Ancient genomic data also allow us to determine the species admixture randomly applied by man during crop cultivation. For instance, if growers cultivated 10 plants belonging to two different but inter-compatible species at the same time, interspecific hybrids between the two species could be generated. Specimen introgressions can only be observed through genome sequencing, which is crucial especially for species that have been widely grown and improved in recent centuries. Large-scale and more in-depth studies using ancient plant genomes can lead to validation or reintroduction of alleles or mutation in modern crops, detected through aDNA sequencing (Figure 1). NGS sequences obtained from aDNA mapped on modern crop genomes with a good coverage can reveal a large number of polymorphisms involved in determining traits of agricultural interest (fruit shape, fruit color, resistance to biotic and abiotic stresses, fruit flavor and so forth). The detected mutations can be recorded *in silico* databases to preserve priceless biodiversity for future generations or reintroduced into modern crops (Figure 1). If the mutations are retrieved in wild relative or cultivated crops, they can be reintroduced with the aid of genomic selection (Bevan et al., 2017). Alternatively, the ancient traits can be recovered by using the latest genome engineering techniques (Andolfo et al., 2016).

Moreover, with the aid of ancient genome sequencing the recent history of local adaptation and improvement of some major crops can be revealed. The production of many crops (whether fresh or processed) has strict regional links worldwide. This can be exemplified by many grape clones (Aversano et al., 2017), Khorasan wheat and other crops (Cooper, 2015). aDNA sequencing can “certify” the genetic correlation between ancient crop remains and local present-day crops, giving added value to produce, whether fresh, or processed, usually highly prized by consumers (Figure 1). This kind of certification is perfectly complementary with modern food tracing methods like bio-markers (Raspor, 2005; Ercolano et al., 2008).

## Conclusions

aDNA genome-wide sequencing studies are achieving greater success thanks to progress in NGS technology. NGS techniques fit well with the fragmented nature of ancient genomes and offer different solutions for a wide range of starting materials and types of studies. The unfathomable genome of ancient crops, concealing extensive potential for modern agriculture, is now accessible. Ancient genomes can shed light on crop evolution and domestication, and also retrieve the history of agriculture in a specific area. Information obtained can be used to steer further research more effectively, aimed at varietal improvement or the management of important crops as well as promoting agricultural products historically connected with a specific area, diet or culture.

## Author contributions

AD was centrally involved in writing the manuscript and producing tables and figures. EF critically revised the manuscript. ME conceived the study, drafted and improved the text. LF coordinated work and contributed to manuscript writing. All of the authors read and approved the final manuscript.

### Conflict of interest statement

The authors declare that the research was conducted in the absence of any commercial or financial relationships that could be construed as a potential conflict of interest.

## Supplementary material

Supplementary Figure 1 Flow chart of aDNA analysis. **(A)** The different sources of aDNA. From left to right: cave and lake sediments, wood remains, spikelets, seeds, fruit, pottery, utensils, herbarium, and human remains. **(B)** Extraction methods. aDNA can be extracted from different starting materials using validated scientific protocols or commercial extraction kits. **(C)** Molecular tools and sequencing approaches. **(D)** Genetic analysis of aDNA. **(E)** Application of aDNA studies.
