# Supplementary figures and images for: Genome Sequencing of Ancient Plant Remains: Findings, Uses and Potential Applications for the Study and Improvement of Modern Crops
Source: Front Plant Sci. 2018 Apr 17;9:441. doi: 10.3389/fpls.2018.00441 (PMC5914272; doi:10.3389/fpls.2018.00441)

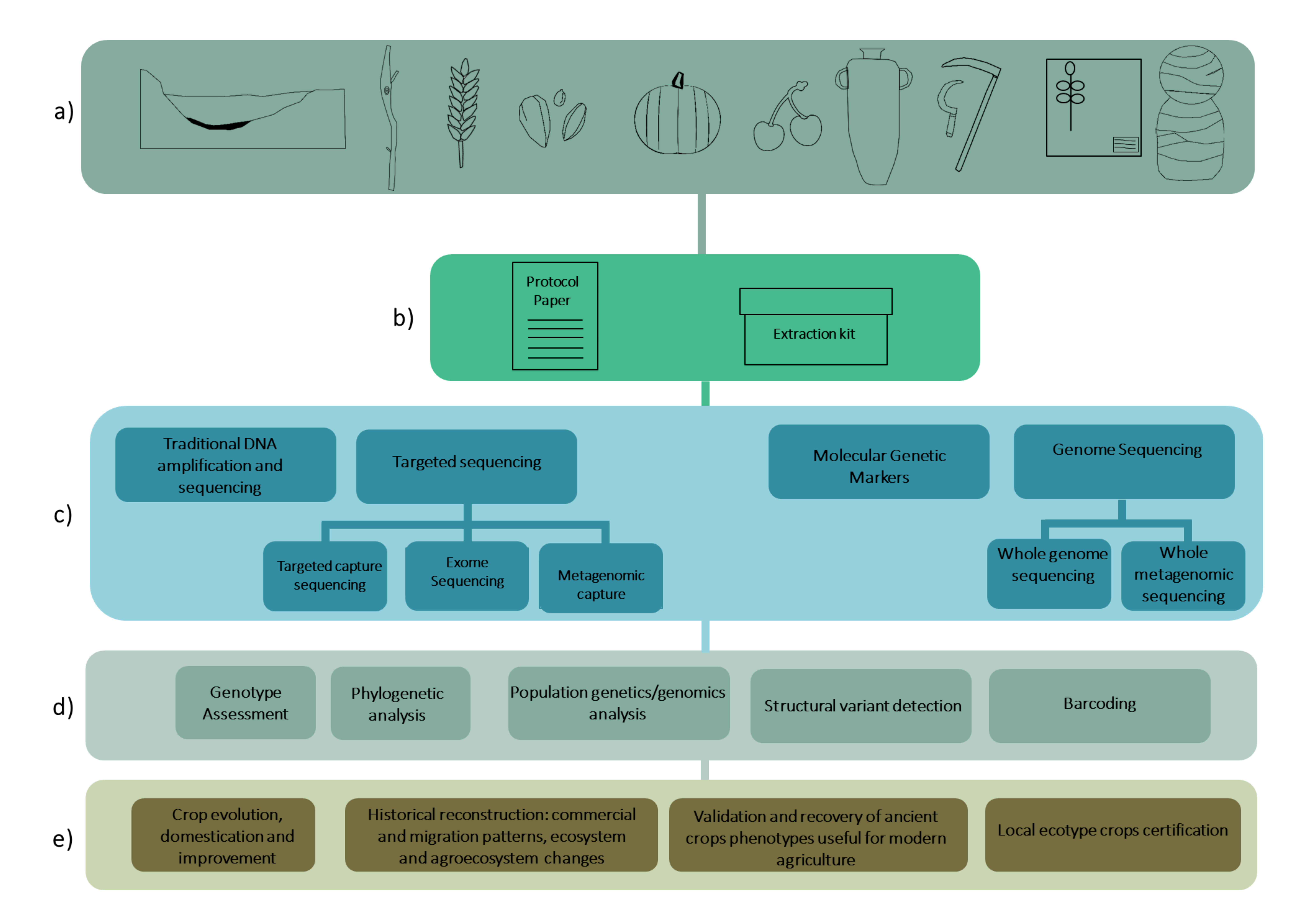

Supplement: Supplementary Figure 1 — Flow chart of aDNA analysis. (A) The different sources of aDNA. From left to right: cave and lake sediments, wood remains, spikelets, seeds, fruit, pottery, utensils, herbarium, and human remains. (B) Extraction methods. aDNA can be extracted from different starting materials using validated scientific protocols or commercial extraction kits. (C) Molecular tools and sequencing approaches. (D) Genetic analysis of aDNA. (E) Application of aDNA studies. [file Image1.tiff]
